# Improving bitter pit prediction by the use of X-ray fluorescence (XRF): A new approach by multivariate classification

**DOI:** 10.3389/fpls.2022.1033308

**Published:** 2022-11-30

**Authors:** Claudia Moggia, Manuel A. Bravo, Ricardo Baettig, Marcelo Valdés, Sebastián Romero-Bravo, Mauricio Zúñiga, Jorge Cornejo, Fabio Gosetti, Davide Ballabio, Ricardo A. Cabeza, Randolph Beaudry, Gustavo A. Lobos

**Affiliations:** ^1^ Plant Breeding and Phenomics Center, Faculty of Agricultural Sciences, Universidad de Talca, Talca, Chile; ^2^ Laboratorio de Química Analítica y Ambiental, Instituto de Química, Pontificia Universidad Católica de Valparaíso, Valparaíso, Chile; ^3^ Facultad de Ciencias Forestales y de la Conservación de la Naturaleza, Universidad de Chile, Santiago, Chile; ^4^ Department of Agricultural Sciences, Universidad Católica del Maule, Curicó, Chile; ^5^ Milano Chemometrics and QSAR Research Group, Department of Earth and Environmental Sciences, University of Milano-Bicocca, Milano, Italy; ^6^ Plant Nutrition Laboratory, Department of Crop Sciences, Faculty of Agricultural Sciences, University of Talca, Talca, Chile; ^7^ Department of Horticulture, Michigan State University, East Lansing, MI, United States

**Keywords:** Malus × domestica, calyx, K/Ca, refrigerated storage, modelling

## Abstract

Bitter pit (BP) is one of the most relevant post-harvest disorders for apple industry worldwide, which is often related to calcium (Ca) deficiency at the calyx end of the fruit. Its occurrence takes place along with an imbalance with other minerals, such as potassium (K). Although the K/Ca ratio is considered a valuable indicator of BP, a high variability in the levels of these elements occurs within the fruit, between fruits of the same plant, and between plants and orchards. Prediction systems based on the content of elements in fruit have a high variability because they are determined in samples composed of various fruits. With X-ray fluorescence (XRF) spectrometry, it is possible to characterize non-destructively the signal intensity for several mineral elements at a given position in individual fruit and thus, the complete signal of the mineral composition can be used to perform a predictive model to determine the incidence of bitter pit. Therefore, it was hypothesized that using a multivariate modeling approach, other elements beyond the K and Ca could be found that could improve the current clutter prediction capability. Two studies were carried out: on the first one an experiment was conducted to determine the K/Ca and the whole spectrum using XRF of a balanced sample of affected and non-affected ‘Granny Smith’ apples. On the second study apples of three cultivars (‘Granny Smith’, ‘Brookfield’ and ‘Fuji’), were harvested from two commercial orchards to evaluate the use of XRF to predict BP. With data from the first study a multivariate classification system was trained (balanced database of healthy and BP fruit, consisting in 176 from each group) and then the model was applied on the second study to fruit from two orchards with a history of BP. Results show that when dimensionality reduction was performed on the XRF spectra (1.5 - 8 KeV) of ‘Granny Smith’ apples, comparing fruit with and without BP, along with K and Ca, four other elements (i.e., Cl, Si, P, and S) were found to be deterministic. However, the PCA revealed that the classification between samples (BP vs. non-BP fruit) was not possible by univariate analysis (individual elements or the K/Ca ratio).Therefore, a multivariate classification approach was applied, and the classification measures (sensitivity, specificity, and balanced precision) of the PLS-DA models for all cultivars evaluated (‘Granny Smith’, ‘Fuji’ and ‘Brookfield’) on the full training samples and with both validation procedures (Venetian and Monte Carlo), ranged from 0.76 to 0.92. The results of this work indicate that using this technology at the individual fruit level is essential to understand the factors that determine this disorder and can improve BP prediction of intact fruit.

## 1 Introduction

Bitter pit (BP) is considered one of the most relevant postharvest disorders in the apple industry worldwide (Al Shoffe et al., 2019). It occurs on a wide geographic range and a significant number of cultivars (Bergmann, 1992; Volz et al., 2006), causing major economic damage to growers and exporters, as it develops progressively during storage (Lötze and Theron, 2006). BP appears mainly in the subepidermal tissue at the calyx end of the fruit, as internal corky lesions (Jarolmasjed et al., 2016; Jemrić et al., 2016). It was first described 150 years ago (Oberdieck and Lucas, 1869), and remains as one of the most studied disorder of apples, except for scald. In general terms, BP is associated with a localized calcium (Ca) deficiency (Perring and Pearson, 1986; Ferguson and Watkins, 1992; Saure, 2005; Fallahi et al., 2006; Fallahi et al., 2010; Sharma et al., 2012; Espinosa-Zúñiga et al., 2017; Torres et al., 2017) in association with an imbalance of other minerals, most frequently nitrogen (N), potassium (K) and magnesium (Mg) (Smock and Van Doren, 1937; Perring and Pearson, 1986; Fallahi et al., 1997; Fallahi et al., 2006; Fallahi et al., 2010; Jarolmasjed et al., 2016; Jemrić et al., 2016; Kalcsits, 2016; Al Shoffe et al., 2019; Kalcsits et al., 2019; Torres et al., 2021). Phosphorus (P), like Ca and Mg, have been found to accumulate in pitted tissue (Chamel and Bossy, 1981).

Ca reaches the roots by mass flow (Jungk and Claassen, 1997) and is translocated to the end of the vascular tissues (shoots, leaves and fruits) by transpiratory flow throughout the xylem (Medrano et al., 2007; López López et al., 2009). Exchangeable soil Ca is rarely responsible for the occurrence of BP, at least not in temperate soils, where it yields more than 50% of exchangeable soil cations (Ca, Mg, K and Na) (Blume et al., 2016). Thus, Ca uptake and subsequent Ca deficiency in fruit is not necessarily a consequence of soil Ca restriction. Consequently, soil applications of Ca have not been as effective in reducing BP in apples as foliar applications (Torres et al., 2017), although in apple orchards planted in acid soils treated with lime or gypsum, a decrease in BP has been observed (Wilms and Basso, 1987). As the fruit develops, the contribution of the xylem to the total inflow to the fruit decreases and the phloem flow increases, implying less Ca delivery to the fruit (Lang, 1990). This loss of xylem functionality, due to the fruit growth, disrupts water mass flow, limiting the transport of Ca to the fruit, which has been suggested to favor development of BP (Alarcón et al., 1998; Miqueloto et al., 2014). Due to this, prediction of BP occurrence by mineralogical analysis in fruit apples is carried out between 40 and 60 days after full bloom, before Ca is diluted up to 50% by fruit growth (Peryea et al., 2007; Miqueloto et al., 2014).

Several factors predisposing and intensifying nutritional imbalances at the limb/fruit level have been associated with BP severity. For example, excessive tree vigor (Terblanche et al., 1979; Baugher et al., 2017) due to an inappropriate rootstock/cultivar combination in response to both soil (Sió et al., 1999; Weibel et al., 2000; Biskup et al., 2003; Fallahi, 2012; Fazio et al., 2013; Sió et al., 2018) and the environmental characteristics (Goode and Ingram, 1971; Bergmann, 1992; Jemrić et al., 2016), as well as the agronomic management that impacts on plant light interception (Van Der Boon, 1980), nutrition (Fallahi et al., 1997; Kim and Ko, 2004), crop load (Ferguson and Watkins, 1992; Volz et al., 1993; Tough et al., 1998; Volz and Ferguson, 1999; Telias et al., 2006; Seo et al., 2007), and fruit ripeness at harvest (Al Shoffe et al., 2020).

Since BP damage often becomes evident after several months of refrigerated storage (Conway et al., 2002; Jemrić et al., 2016) and control methods are not always effective (Fernández et al., 2009), significant efforts have been committed to developing methodologies for early prediction of the disorder. Fruit mineral analysis and infiltration by Mg salts remain the most used predictive tools, but with different levels of effectiveness (Baugher et al., 2017). The former, is used to estimate Ca content as well as its relationship with other nutrients. In general this method has a low predictive capacity (10 – 40%) given the need to compose the sample with more than one fruit (Al Shoffe et al., 2019). Nevertheless, in a study where the mineral concentration of the fruit was determined three weeks before commercial harvest, it was concluded that (K+Mg)/Ca, (N+K+Mg)/Ca or N/Ca ratios were highly correlated with the occurrence of BP in ‘Honey Crisp` apples, which is a very susceptible cultivar (Marini et al., 2020). The main disadvantage is the analysis based on a group of fruits, that increases variability on the results. On the other hand, infiltration by Mg salts has a more significant association with BP (<70%), but is time consuming, destructive and difficult to implement massively (Bangerth, 1974; Hopfinger and Poovaiah, 1979; Burmeister and Dilley, 1991; Retamales et al., 2000; Retamales et al., 2001; Amarante et al., 2010; Torres et al., 2015).

The relevance of the relationship between K and Ca is well known, and is of particular interest for the present study. For example, Val et al. (1999) reported that the risk of BP increased considerably when, between 80 – 100 d after full bloom, the ratio in fruit increased above 25 and in leaf fell below two. Today, K/Ca remains as a reference for any cultivar anywhere in the world (Lötze et al., 2008; von Bennewitz et al., 2015; Espinosa-Zúñiga et al., 2017). Recently, Prengaman (2021) proposed, as indicator of BP occurrence, the nutrient content of apple juice, indicating that K/Ca ratio explained about 70% of the variation in BP of ‘Honey Crisp’ samples. Although the K/Ca ratio could provide valuable information for the industry, a high variability of Ca and K content has been reported within the fruit as well as between fruits on a plant (Wills et al., 1976; Ferguson and Triggs, 1990; Le Grange et al., 1998; Lötze and Theron, 2006).

Therefore, it is pertinent to study the problem at the individual sample level and, ideally, using non-destructive tools. Although some non-destructive approaches are not able to determine the nutrient content (i.e. non-mineral approach), they can estimate it. In this sense, proximal and non-proximal remote sensing equipment, such as VIS/NIR/SWIR spectroscopy (i.e., ~350 – 2,500 nm) are widely used to estimate the nutritional status of plant tissues (Nicolaï et al., 2006; Kafle et al., 2016; Jarolmasjed et al., 2017; Jarolmasjed et al., 2018; Mogollón et al., 2021), mainly in leaves but also in other organs (García-Sánchez et al., 2017). For example, spectral reflectance at fruit level was useful in identifying BP-fruit, however, other lesions were misclassified as BP (Nicolaï et al., 2006; Jarolmasjed et al., 2017).

Less developed in fruit research, X-ray fluorescence (XRF) spectroscopy was designed for the semi-quantitative measurement of mineral levels in different materials, thus with potential to determine mineral elements and their ratios (e.g., K/Ca) (Espinosa-Zúñiga et al., 2017). Handheld XRF devices accurately quantify the photon emission of elements with an atomic weight greater than 28.1 (i.e., silicon, Si), which includes Ca and K. Kalcsits (2016) demonstrated that XRF could be used for non-destructive semi-quantitative determination of Ca and K in apples and pears, and there was a significant high correlations with mineralogical content coming from traditional laboratory analysis; however, like others (Mohr and Jamieson, 1984; Baugher et al., 2017), this work highlights that beyond K/Ca, other elements are implicated in the occurrence of the disorder. Therefore, it is hypothesized that using a multivariate modeling approach, other elements in addition to K and Ca could be found to improve the current predictive capability of BP.

## 2 Material and methods

### 2.1 Apple fruit used for bitter pit prediction

Two studies were carried out as follows:

#### 2.1.1 Study 1

**Figure 1:**
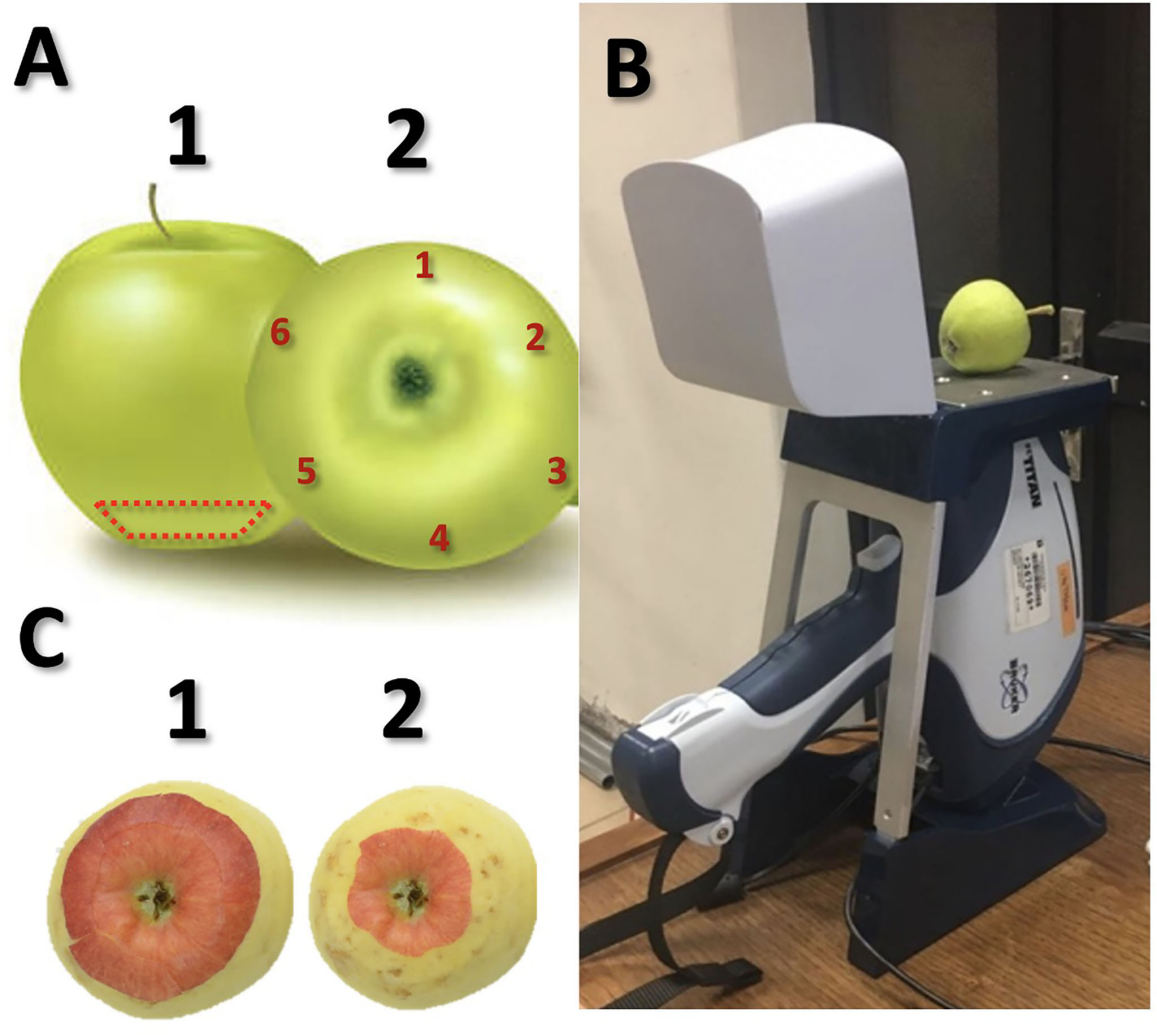
Section of the fruit calyx (dashed red line) where the bitter pit is usually located **(A-1)** and where the reproducibility XRF study was performed at six equidistant points **(A-2)**; details of XRF measurement **(B)**; bitter pit in fruit with no external symptoms, before **(C-1)** and after fruit peeling **(C-2)**.

**Figure 2:**
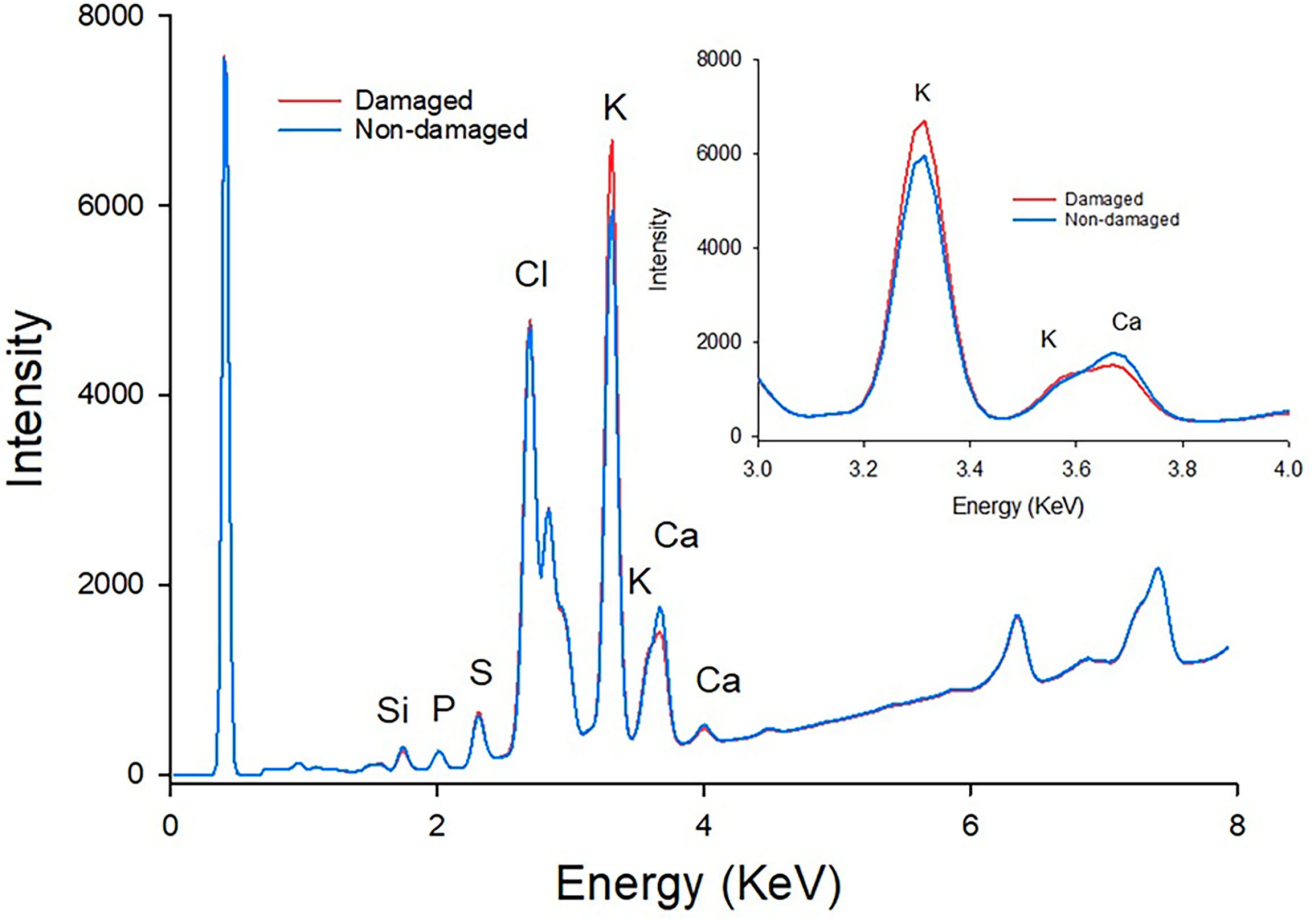
General XRF spectra for ‘Granny Smith’ apple epidermis for fruit with (red) and without (blue) bitter pit; K and Ca peaks details in small graphic. Similar spectra were found in the other cultivars.

A balanced database was generated with apples with and without BP symptoms. For this purpose, during the 2018/19 season, Dole Chile Co. provided two groups of ‘Granny Smith’ apples from a commercial lot of refrigerated storage (5 months at 0°C and 90% RH): i) lot 1 with BP: 176 apples with medium and severe epidermal incidence of BP (i.e., 3 – 5, and >5 pits, respectively); and ii) lot 2 without BP: 176 apples with no evidence of BP injury (i.e., no epidermal indication of BP or other disorder). Since most of the BP damage is concentrated towards the distal region of the fruit, the evaluations were concentrated in the calyx area (**Figure 1A-1**). To determine the variability associated with XRF measurements at fruit level, a reproducibility study was conducted, in which six equidistant points were measured along the calyx end of each pitted and non-pitted fruit (**Figure 1A-2**).

#### 2.1.2 Study 2

To predict the occurrence of BP, apples harvested from a commercial orchard were used. The fruit were collected during the 2019/20 season from two orchards with previous records of medium to high BP incidence. The orchards are located in the Maule Region – Chile: i) San Clemente (35°30’52” S; 71°26’42.8” W): ‘Granny Smith’/MM9 and ‘Brookfield’/MM9, planted in 2011 (3.5 x 1.2 m; NW/SE orientation); and ii) Lontue (35°15’39” S; 71°14’32.0” W): ‘Fuji’/MM9, planted in 2007 (4.5 x 1.5 m; NW/SE orientation). Trees were trained under Solaxe system, with three distinguishable productive floors, having the upper third a lower amount of fruit and leaves compared to the inferior floors (i.e., lower and middle); the insertion of each branch into the trunk determined the floor to which fruit corresponded. Given that, in previous seasons, more than 70% of the BP (developed after storage) was found in the upper floor of the trees in these orchards (data not shown), during the 2019/20 season, all fruit from the upper floor of ten homogeneous trees of each orchard and cultivar, were collected at harvest. The fruit were transferred to the Postharvest Laboratory of the Universidad of Talca where elemental composition was determined with a handheld XRF device by measuring the calyx area of each fruit (**Figure 1**). After this, apples were stored for 5 months at 0°C and 90% RH to induce BP appearance. Upon storage removal, fruit were placed at room temperature at 20°C for 10 d to accelerate the expression of the disorder. The incidence and severity of BP were visually scored and classified (**Figure 1B**) as undamaged (i.e., without evident damage inside and outside the fruit) and damaged fruit (i.e., mild, medium, and severe BP, consisting of<2; 3 – 5, and >5 pits, respectively), by peeling all fruit at the calyx end.

The operation condition of the XRF instrument (Titan S1, Bruker Elemental portable handheld X-ray fluorometer, Kennewick, WA, USA) was a voltage of 21 kV, amperage of 40 µA, and 15 s of exposure. As proposed by Kalcsits (2016) to ensure that as many X-rays bombard the sample, each apple was placed with as much contact as possible with the instrument’s surface (**Figure 1C**). Since the device is sensitive to X-rays with photon energies above 1.5 keV, it is capable of simultaneously determining chemical elements heavier than silicon (Si). Consequently, elements other than K and Ca were studied at the same time; between 1.5 and 8 keV. The estimated penetration depth of X-rays is approximately 1 mm, and therefore, the elemental composition of skin and flesh was determined (Kalcsits, 2016).

### 2.2 Statistical data analysis and modelling of the XRF signature

#### 2.2.1 Ionomics characterization of ‘Granny Smith’ fruit with BP (Study 1)

For the exploratory analysis of the complete XRF spectra, a Principal Component Analysis (PCA) was carried out. The main objective of PCA is to reduce many variables to a smaller set of factors, named principal components (PCs) while retaining most of the information from the original dataset (Wold et al., 1987; Bro and Smilde, 2014). Briefly, the data matrix *X* (with *n* samples and *m* variables) was decomposed according to equation (1), where *T*_(_ *_n,A_*_)_ is the score factor matrix and *P*_(_ *_m,A_*_)_ is the loading factor matrix for *A* components.


Equation (1) $$X_{\left(n,m\right)}=T_{\left(n,A\right)}\cdot P_{\left(A,m\right)}^{T}$$

In this model, the scores represent the projection of *n* samples on the reduced subspace of *A* dimensions, where *A*< *m*. The critical parameters to be adjusted are the number of principal components (PCs) and the preprocessing data analysis. In this study, the number of PCs were determinate by full-cross validation strategy and the preprocessing procedures considered were mean-centering and autoscaling of spectral data.

For a quantitative understanding of such differences between both BP groups the signal intensities of each element were compared by ANOVA, cumulative frequency distribution and box-and-whisker plots. For the particular case of K/Ca, two approaches were developed: i) deconvolution of the K and Ca peaks and subsequent calculation of the ratio (Kalcsits, 2016); and ii) simple ratio, where the values measured by the equipment were used directly (i.e., K and Ca without deconvolution).

#### 2.2.2 Prediction of BP by multivariate XRF signature modeling (Study 2)

For classification purposes of the fruit coming from orchards with previous records of medium to high BP incidence, Partial Last Squares Discriminant Analysis (PLS-DA) was considered. The principal advantage of this method is to combine the dimensionality reduction and discriminant analysis into one algorithm, being especially useful to analyze high dimensional data (Lee et al., 2018). Basically, a PLS-calibration model is built between one *X_(n,m)_* data matrix and the *Y_(n,1)_* vector containing the class labels of samples. The *X_(m,n)_* data matrix is decomposed through the PLS algorithm that searches for latent variables (LVs) with a maximum covariance with the Y-variable. Since predicted values are quantitative, then samples can be classified according to a rule, such as the labelling to the class corresponding to the highest prediction. More detailed and formal description of the algorithm is presented in a previous publication (Barker and Rayens, 2003).

All data were analyzed by using commercial software (Matlab, Mathworks Inc., MA, USA). For PCA and PLS-DA, procedures were calculated by means of Guide to User Interface (GUI) available in internet (https://michem.unimib.it/, last revision: 30 March 2022) and written in MATLAB language (Ballabio and Consonni, 2013; Ballabio, 2015).

Prior to supervised classification, data were explored by means of PCA to remove anomalous samples, which were identified based on extreme values of Q residuals and Hoteling T^2^ (Bro and Smilde, 2014). The retained spectra were subjected to PLS-DA models on autoscaling data and applied to classification of samples classified in two groups: affected and non-affected by BP. The best classification models were obtained when fruit on the affected group excluded the mild category (i.e., only apples classified as moderate and severe incidence were considered).

The performance to distinguish BP and non-BP fruits was evaluated using the following classification measures (Ballabio et al., 2018): sensitivity (*Sn*, ratio of correctly classified non-BP samples), specificity (*Sp*, ratio of correctly classified BP samples), and balanced accuracy *(BA)*, which is the average of sensitivity and specificity. Classification models were validated through two different approaches: i) internal cross-validation (5 groups split with the venetian blinds procedure); and ii) Montecarlo validation based on 100 iterations (in each iteration 20% of samples were randomly selected and used to test the classification models).

## 3 Results

### 3.1 Study 1: Determination of the K/Ca ratio by XRF on fruit with and without bitter pit

When the balanced data base of ‘Granny Smith’ apples with and without BP was studied, the X-ray spectra (**Figure 2**) showed that the most significant peaks corresponded to potassium (K: 3.31 and 3.59 keV), calcium (Ca: 3.69 and 4.01 keV), and chloride (Cl: 2.68 and 2.82 keV); less intense peaks were found for silicon (Si: 2.02 and 2.46 keV), phosphorus (P: 2.02 and 2.14 keV), and sulfur (S: 2.31 and 2.46 keV). For these spectra, the most affected samples exhibited higher K intensity, while non-affected apples showed higher Ca intensity (**Figure 2**).

**Figure 3:**
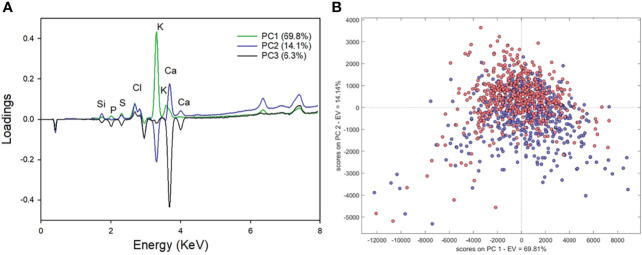
Loading **(A)** and score **(B)** plots obtained with XRF-spectra of ‘Granny Smith’ apples with (blue) and without (red) bitter pit.

Effectively, the analyzed spectra by a PCA model showed that K (3.31 keV) and Ca (3.69 keV) peaks contributed with the maxima variability for each component (**Figure 3A**). The dispersion of these two signals, evaluated as standard deviation and coefficient of variation, are shown in **Supplementary Table 1**. The results evidenced that the random error for K and Ca responses varied in the range of 10 to 25% (data not shown); considering that instrument variation is lower than 10%, this dispersion could be associated to heterogeneity within the apple composition. Additionally, the score plot (**Figure 3B**) obtained after PCA analysis shows a severe overlapping between samples with and without BP, suggesting an important similarity between spectral signature of the fruits.

**Figure 4:**
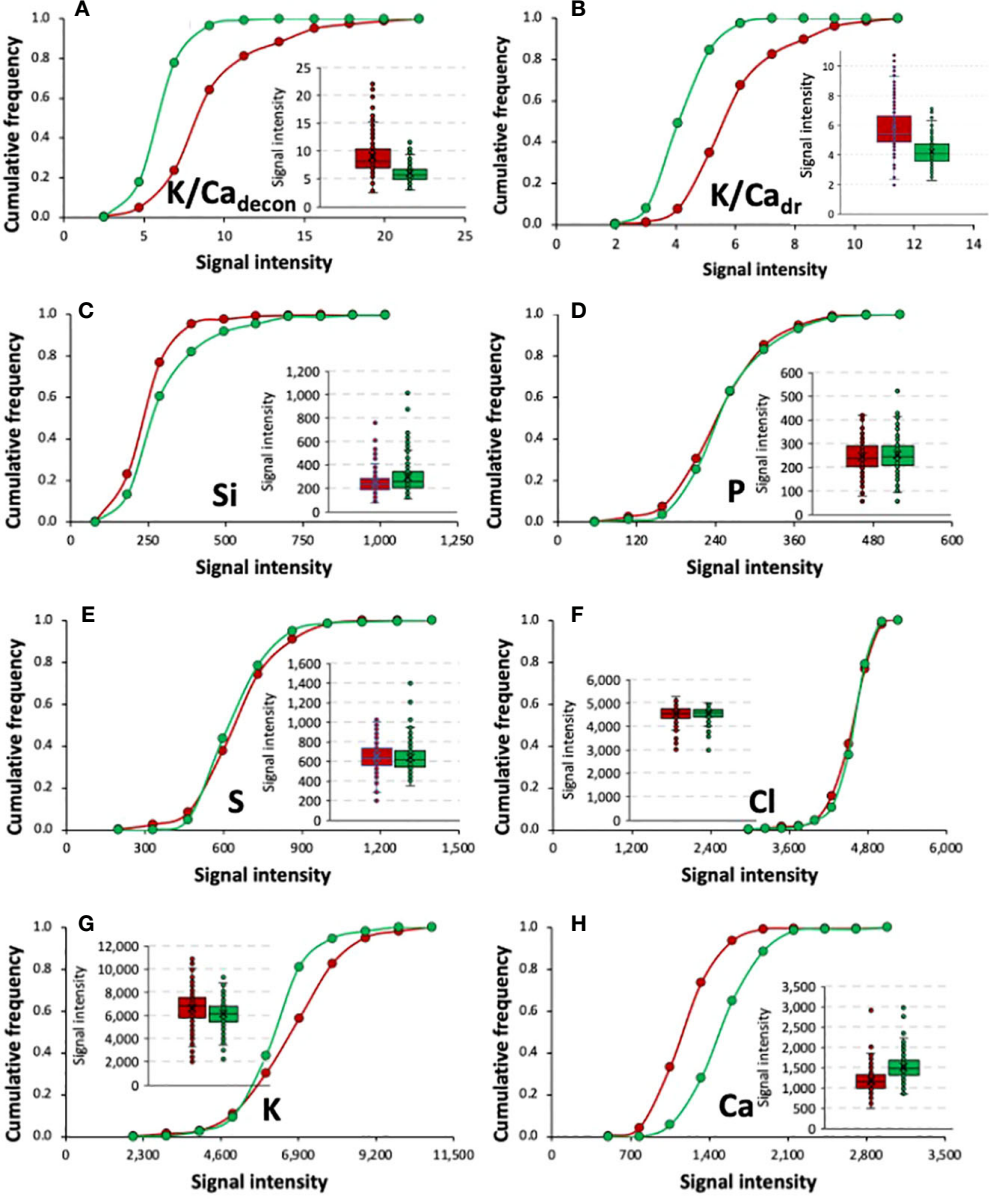
Comparison of the cumulative frequency distributions and box-and-whisker plots of K/Ca ratio calculated by the deconvolution method (decon) **(A)** and direct ratio (dr) **(B)** from maximum K and Ca signals, and the signal insensitivity of the mineral elements with the greatest preponderance in ‘Granny Smith’ fruit with (red) or without (green) bitter pit: Si **(C)**, P **(D)**, S **(E)**, Cl **(F)**, K **(G)**, and Ca **(H)**.

Especially for the K/Ca ratio calculated after the element deconvolution of the data matrix of ‘Granny Smith’, the results showed a high variability in sound fruits (~5 – 23), differences that almost doubled in the case of affected ones (the ratio ranged from ~4 – 41) (**Supplementary Table 1**). Globally, 80% of the fruit without BP had a K/Ca ratio of less than ~7, while in those with BP, for the same ratio, the proportion was less than 24% (**Figure 4A**); although with different scales, similar results were found for K/Ca (**Supplementary Table 1**, and **Figure 4B**). From same data base, the analysis of variance (**Supplementary Table 1**) the cumulative frequency distribution and the box-and-whisker plots (**Figures 4C–H**) of all potential identified elements indicated that, except for Cl and partially for P (p=0.0931), the rest of the elements differed between BP groups (p≤ 0.00001). On the other hand, from a methodological point of view, no differences were found among the six equidistant points at the calyx-end for any of the elements demonstrating that measurements at the calyx is consistent. Furthermore, no significant interactions were found between the two factors, indicating that the main difference between samples was due to the BP presence/absence (**Supplementary Table 1**). K/Ca ratio significance was the same regardless of the way in which they were calculated (deconvolution method *vs* direct ratio from maximum K and Ca signals); also, a high association (R^2^ = 0.98) was found between both (**Supplementary Figure 1**).

The best PCA models were obtained after mean-centering and using 4 or 5 components to reach explained variances higher than 90%. As observed in **Figure 3**, the score plot resulted in 69.8% of explained variance for PC1 and 14.1% for PC2 (**Figure 3A**) and ~90% when PC3 was included. The more explicative spectral region considers the characteristic region of K, Ca, and Cl (i.e., 2.6 to 3.7 keV). Nevertheless, a significant influence is observed for lower energy signals between 1.7 and 2.5 keV, typical for Si, S, and P.

### 3.2 Study 2 (field trials): Multivariate classification of bitter pit disorder by using XRF spectra on three apple cultivars

All fruit from the upper section of the tree was harvested and initially measured by XRF; from these samples BP developed on 18.4, 3.6, and 2.4% of ‘Granny Smith’, ‘Brookfield’ and ‘Fuji’ apples, respectively (average of ten trees), as detailed on **Table 1**. For the XRF analyses, the spectra of the apples harvested in the field (‘Brookfield’ and ‘Fuji’) (**Supplementary Figure 2**) were similar to those of the records generated by the ‘Granny Smith’ balanced database (section 3.1), so they would provide similar information and therefore be consistent with the objectives of the multivariate modeling.

**Table 1:** Number of total fruits used for ‘Granny Smith’-GS, ‘Brookfield’-BF and ‘Fuji’-FJ apples coming from commercial orchards for BP prediction.

Cultivar	Total number of harvested fruits	Fruit within each class after storage		
		Without BP	With BP	% of BP fruit
GS	5267	4296	971	18.4
BF	4375	4126	159	3.6
FJ	2899	2828	71	2.4

**Figure 5:**
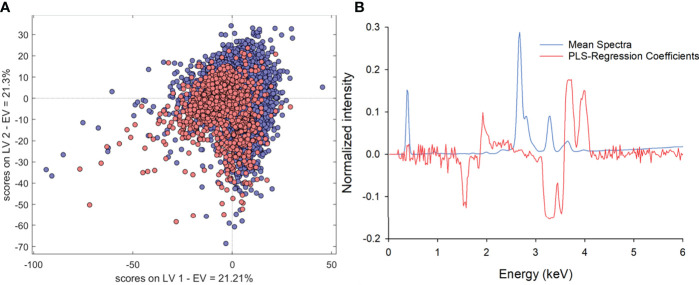
Score **(A)** and normalized intensity **(B)** plots for ‘Granny Smith’ apples coming from field trials, comparing non affected (blue) and affected (red) fruit.

In the same way as described for ‘Granny Smith’ apples on section 3.1, the classification between samples (BP vs. non-BP fruit) was not possible by univariate analysis (individual elements or the K/Ca ratio). Therefore, multivariate approaches, using a selected spectra region (1.5 to 4.95 keV), were evaluated. A preliminary estimation showed that the classification of the different damage levels on apple samples was not possible by using linear (LDA, SIMCA and PLS-DA) and nonlinear (QDA and NNA) multivariate models due to severe overlapping of the different classes. For this reason, the classification between two groups: damaged- (including moderated and severe damage) vs. undamaged-fruit was considered for further analysis. This approach allowed to increase the balance of data set (samples with severe BP are less abundant) and improved the evaluated classification models. From the several multivariate tested methods, the best results were obtained with PLS-DA. For this, auto scaled preprocessing and 3 to 4 components were required to explain over 80% of the variance. The PLS-DA-scores obtained for ‘Granny Smith’ suggest a better separation between sound and affected fruit (**Figure 5A**). In addition, the higher PLS-DA-regression coefficients are obtained for first 300 channels, corresponding from 1.5 up to 4.95 keV (**Figure 5B**).

As expected, the more explicative spectral region considers the characteristic section of K, Ca, and Cl (i.e., 3.0 to 4.0 keV). Nevertheless, a significant influence is observed for lower energy signals between 1.5 and 2.5 keV, typical for Si, S, and P. The same consideration can be extended to the other cultivars because similar results were obtained. Sensitivity, specificity, and balanced accuracy of PLS-DA models for all cultivars, evaluated on the full training samples and with both validation procedures (venetian blinds and Montecarlo), ranged from 0.76 to 0.92 (**Table 2**).

**Table 2:** Classification measures (balanced accuracy, specificity, and sensitivity) obtained in fitting, cross validation (with 5 groups split in Venetian blinds and Montecarlo approach) for the three apple cultivars (‘Granny Smith’-GS, ‘Brookfield’-BF, and ‘Fuji’-FJ).

		Training samples			Cross validation(5-fold)			Montecarlo validation(20% out)		
Cultivar	LVs	BA	Sn	Sp	BA	Sn	Sp	BA	Sn	Sp
GS	4	0.78	0.77	0.79	0.77	0.76	0.77	0.76	0.77	0.76
BF	4	0.84	0.82	0.87	0.82	0.82	0.82	0.81	0.82	0.80
FJ	3	0.87	0.83	0.92	0.81	0.84	0.77	0.81	0.83	0.79

PCs, PLS-DA Latent variables. BA, average of sensitivity and specificity; Sn, Sensitivity; Sp, Specificity.

## 4 Discussion

Establishing a fruit orchard implies many agronomic decisions (e.g., site selection, rootstock, cultivar, planting density, row orientation, training system, irrigation system) that, along with practical managements (nutrition, crop load, pruning, pollination), will affect both, the vegetative and reproductive expression of the plant; thus, also the incidence of physiological disorders as BP. In the orchards of the present experiment, the BP was found mainly on the upper production floor, where there was a lower amount of fruit and leaves compared to the lower floors.

In older orchards, with planting frames such as the one studied (i.e., 3.5 – 4.5m x 1.2 – 1.5 m), if there is no reasonable control of vegetative expression, it is common to find an excess of leaves and an uneven distribution of the leaf/fruit ratio within the plant. Since Ca movement within the plant occurs through transpiration flow, the number and distribution of leaves within the canopy should also influence the location and accumulation of the element. Thus, because microclimatic characteristics at each plant floor or branch section vary between days (e.g., maximum water pressure deficit, cloudiness, and incident radiation) and throughout the season (e.g., translational effects of the sun), the location of the clutter is difficult to predict (Baugher et al., 2017). In the present study, the upper part of the tree would be subjected to a more prolonged daily radiative stress than in the lower floors, making Ca supply to that section of the tree even more challenging, thus predisposing its fruits to develop the disorder.

In the present study for all cultivars, the strongest signals in the XRF spectrum (**Figure 2**) corresponded to K and Ca. Although the K/Ca ratio is the most cited proxy related to the disorder, BP has been characterized as an alteration of a more complex ionic content of the damaged tissues, affecting other elements that are involved in essential physiological processes (Simons and Chu, 1980; Chamel and Bossy, 1981; Garrec, 1983; Baugher et al., 2017). Probably because of that, quite often, predictions based on such ratio can be erratic, as lots with relatively high Ca concentration (above 5 mg Ca 100 g^-1^ FW) may end up developing BP, whereas fruit below that threshold don’t always do (Terblanche et al., 1980; Le Grange et al., 1998; Lötze and Theron, 2006). The range of values on the elements found in fruit with and without BP (both as cumulative frequency distributions and box-plots, **Figure 3**), also evidenced this trend, indicating that classification of the disorder is not possible using an univariate approach (**Figure 3B**). Nevertheless, because of all above discussed, more than the known relevance of the relationship between K and Ca (i.e., fruits with BP symptoms use to have higher K/Ca than those without BP), there is a need to study the ionomics of the disorder, but at the individual fruit level.

In this sense, along with K and Ca, other four elements proved to be involved in the occurrence of BP (i.e., Cl, Si, P, and S) (**Figure 3A**). As it can be seen from PCA the first component (i.e., K, Ca, and Cl peaks) indicates that all the samples with severe BP show a major content of these elements. In contrast, the second component has a negative influence of Cl and a positive influence of Ca and K. In addition, the influence of less abundant elements, such as Si (1.74 and 1.83 keV) and S (2.31 and 2.46 keV) appear significant for the second and third components, suggesting a more complex relationship between BP and fruit mineral composition. Similar, Chamel and Bossy (1981) reported a high variability of P and Cl in apples with and without BP, and a lower impact of S. Likewise, Ca, K, Mg, and P concentrations were higher in those apples that presented the disorder (Garrec, 1983; Al Shoffe et al., 2014). Baugher et al. (2017) developed multiple regression models for BP prediction in ‘Honey Crips’ apples, indicating that the best two-variable-model included N/Ca ratio and shoot length (R^2^ = 0.68); when P, boron (B), S, Ca, and Mg/Ca were added to the model, the coefficient of determination improved (R^2^ = 0.71). More recently, Marini et al. (2020) evaluated different models to predict BP on 17 `Honeycrisp´ apple orchards, concluding that the two-variable model including B and Mg/Ca ratio was the best (R^2 =^ 0.83), however, it seems to be not conclusive since in previous season the best model containing N/Ca ratio and the shoot length underestimate the BP incidence.

Despite the ability of the multivariate PCA model to identify Si, P, S, Cl, K and Ca as potential elements involved in the disorder, there was no clear differentiation (i.e., strong data overlapping) between BP severity samples (none, moderate and severe symptoms, **Figure 3B**). Nevertheless, when multivariate classification approaches (i.e., contrasting fruit with vs. without BP) were considered, discrimination between sound and BP affected fruit improved significantly; for all cultivars, the sensitivity and specificity were higher than 0.76 for the training set, demonstrating that the models allowed to correctly recognize at least 78% of the samples affected by BP and reject the class “damaged” for at least the 76% of non-affected samples. In addition, the balanced accuracy showing a similar result, evidenced that the calibration model classifies correctly over 77% of analyzed samples.

Finally, to improve the level of BP prediction, using this non-destructive method, fruit could be examined periodically during the season to determine their elemental content. This is special important to supervise the effectiveness of Ca foliar applications. In addition, due to the advancement of XRF technologies, new instruments allow determining lighter elements such as N, B and Mg (micro-XRF) which could further improve the predictive power, considering their potential role on BP development.

## 5 Conclusions

The XRF reproducibility study in the calyx area of apple shows the potential of this tool for the development of ionomics at the individual fruit level. As a practical example, the easy calculation of K/Ca compared to the deconvolution process of K and Ca peaks would allow simplified monitoring of the K/Ca ratio under field conditions.

As discussed, given the infinite combinations of factors that influence the occurrence of BP (i.e., plant, climate, and management), it isn’t easy to venture which branch or part of it will produce fruit with more or less BP. Given the potential that classification methods would have for BP prediction, combining other databases that allow a more holistic understanding of the problem (e.g., plant reflectance) is relevant.

## Data availability statement

The raw data supporting the conclusions of this article will be made available by the authors, without undue reservation.

## Author contributions

CM, SR-B, MZ, RB, and GL contributed to the conception and design of the work. FG, DB, RC, RBe, JC and MB performed field experiment, acquisition, analysis, and interpretation of data for the work. CM, SR-B, MZ, RB, MB, and GL, collaborated to generate and validate the version to be published.

## Funding

RB acknowledges support from Michigan State University (MSU) AgBioResearch, and the USDA National Institute of Food and Agriculture. In Chile, this work was funded by the National Agency of Research and Development (ANID; FONDEF ID18I10214) and FONDEQUIP EQM200239.

## Acknowledgments

The authors sincerely thank DOLE Chile and Central Frutícola San Clemente for the supply of fruit and the permanent technical support.

## Conflict of interest

The authors declare that the research was conducted in the absence of any commercial or financial relationships that could be construed as a potential conflict of interest.

## Publisher’s note

All claims expressed in this article are solely those of the authors and do not necessarily represent those of their affiliated organizations, or those of the publisher, the editors and the reviewers. Any product that may be evaluated in this article, or claim that may be made by its manufacturer, is not guaranteed or endorsed by the publisher.
