# Supplementary figures and images for: Improving bitter pit prediction by the use of X-ray fluorescence (XRF): A new approach by multivariate classification
Source: Front Plant Sci. 2022 Nov 30;13:1033308. doi: 10.3389/fpls.2022.1033308 (PMC9748620; doi:10.3389/fpls.2022.1033308)

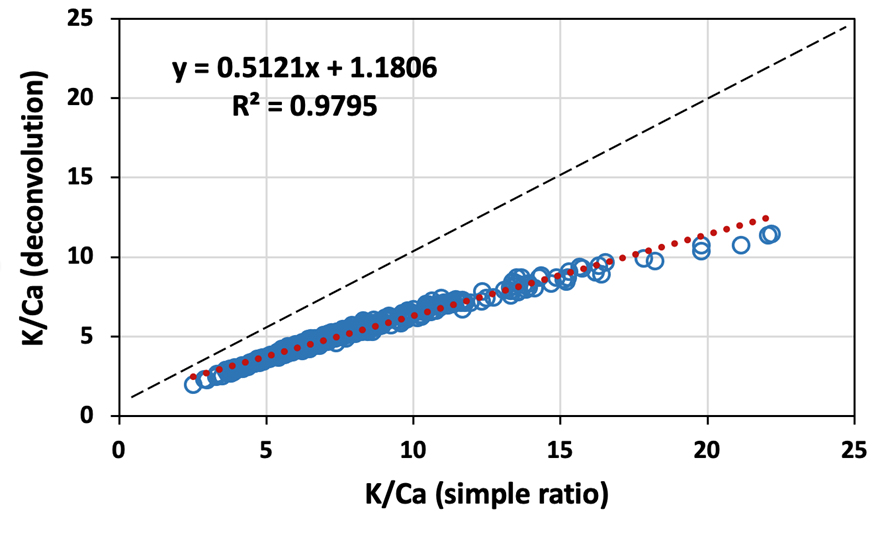

Supplement: Supplementary file 1 [file Image_1.jpeg]

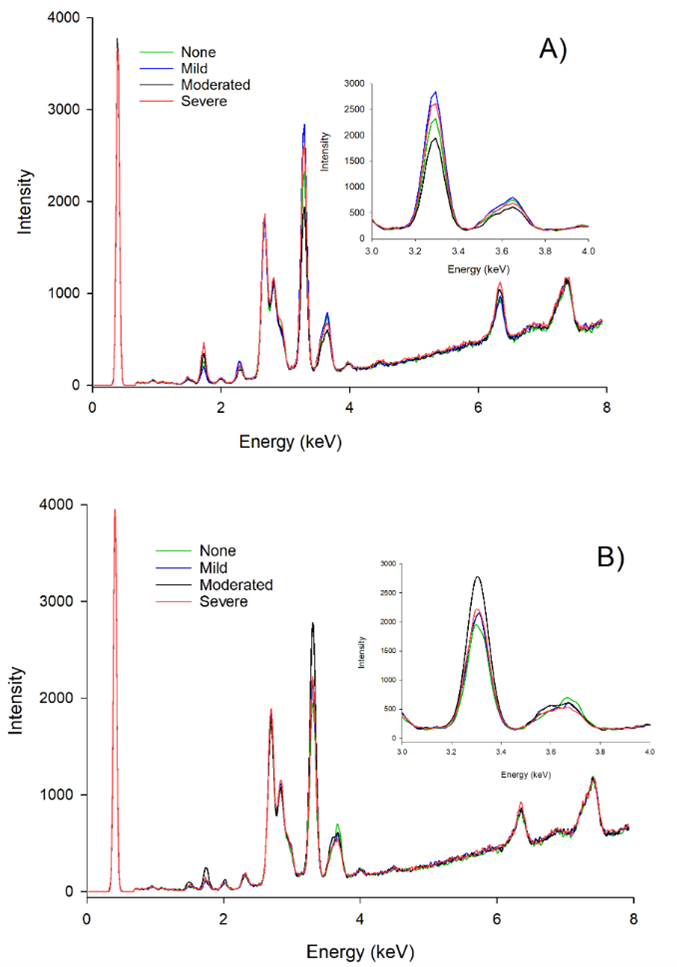

Supplement: Supplementary file 2 [file Image_2.jpeg]
