# Supplementary material for: Improving bitter pit prediction by the use of X-ray fluorescence (XRF): A new approach by multivariate classification
Source: Front Plant Sci. 2022 Nov 30;13:1033308. doi: 10.3389/fpls.2022.1033308 (PMC9748620; doi:10.3389/fpls.2022.1033308)
Supplement: Supplementary file 3 [file Table_1.docx]

**Supplementary Table**

**Supplementary Table 1.** P-values of ANOVA associated with calyx position (the position of XRF sensor at the fruit level, at six equidistant points) and bitter pit incidence (with or without) in 'Granny Smith' apples. Statistical parameters for the signal of the elements (and respective XRF channel) found in fruit with and without bitter pit.

| **ANOVA** | **Si** | **P** | **S** | | **Cl** | **K** | **Ca** | **K/Ca** | |
| --- | --- | --- | --- | --- | --- | --- | --- | --- | --- |
|  | (87) | (102) | (117) | | (135) | (167) | (186) | Deconvolution | Simple ratio |
| Calyx position (CP) | 0.8957 | 0.9154 | 0.9706 | | 0.4174 | 0.7916 | 0.4434 | 0.9607 | 0.9524 |
| With or without bitter pit (BP) | 0.0000 | 0.0931 | 0.4362 | | 0.5085 | 0.0000 | 0.0000 | 0.0000 | 0.0000 |
| CP x BP | 0.8030 | 0.6787 | 0.8865 | | 0.1794 | 0.6686 | 0.5372 | 0.9999 | 0.9990 |
| **Without BP** | | | | | | | | | |
| Count | 264 | 264 | 264 | 264 | | 264 | 264 | 264 | 264 |
| Average | 296.7 | 253.5 | 636.6 | 4541.9 | | 6176.5 | 1507.3 | 9.90 | 4.20 |
| Standard deviation | 137.6 | 64.8 | 135.5 | 287.1 | | 1075.5 | 305.8 | 2.87 | 0.89 |
| Coefficient of variation (%) | 46.4 | 25.6 | 21.3 | 6.3 | | 17.4 | 20.3 | 29.0 | 21.2 |
| Minimum | 111 | 57 | 349 | 2978 | | 2224 | 844 | 4.56 | 2.26 |
| Maximum | 1016 | 521 | 1398 | 5013 | | 9383 | 2982 | 22.75 | 7.23 |
| Range | 905 | 464 | 1049 | 2035 | | 7159 | 2138 | 18.19 | 4.97 |
| Standard skewness | 12.98 | 3.83 | 8.58 | -12.44 | | 0.49 | 4.73 | 7.47 | 4.65 |
| Standard kurtosis | 16.88 | 3.69 | 13.68 | 21.51 | | 4.19 | 7.32 | 6.31 | 1.89 |
| **With BP** | | | | | | | | | |
| Count | 264 | 264 | 264 | 264 | | 264 | 264 | 264 | 264 |
| Average | 243.6 | 243.9 | 646.3 | 4524.5 | | 6653.6 | 1179.2 | 15.85 | 5.85 |
| Standard deviation | 88.6 | 66.9 | 146.6 | 315.8 | | 1440.8 | 277.8 | 6.34 | 1.62 |
| Coefficient of variation (%) | 36.4 | 27.4 | 22.7 | 7.0 | | 21.7 | 23.6 | 40.0 | 27.7 |
| Minimum | 78 | 56 | 197 | 3013 | | 1997 | 501 | 3.73 | 1.96 |
| Maximum | 758 | 420 | 1043 | 5266 | | 10868 | 2917 | 40.79 | 11.46 |
| Range | 680 | 364 | 846 | 2253 | | 8871 | 2416 | 37.06 | 9.50 |
| Standard skewness | 10.34 | 0.69 | 0.96 | 8.57 | | -0.99 | 8.16 | 8.94 | 6.02 |
| Standard kurtosis | 17.96 | 0.74 | 1.90 | 11.69 | | 2.20 | 17.40 | 7.22 | 3.46 |
